# The Inhibitory Effect and Adsorption Properties of Testagen Peptide on Copper Surfaces in Saline Environments: An Experimental and Computational Study

**DOI:** 10.3390/molecules30153141

**Published:** 2025-07-26

**Authors:** Aurelian Dobriţescu, Adriana Samide, Nicoleta Cioateră, Oana Camelia Mic, Cătălina Ionescu, Irina Dăbuleanu, Cristian Tigae, Cezar Ionuţ Spînu, Bogdan Oprea

**Affiliations:** 1 Faculty of Sciences 107i Calea Bucuresti, Department of Chemistry, University of Craiova, 200478 Craiova, Romania; aurelian.dobritescu@edu.ucv.ro (A.D.); catalina.ionescu@edu.ucv.ro (C.I.); irina.dabuleanu@edu.ucv.ro (I.D.); cristian.tigae@edu.ucv.ro (C.T.); cezar.spinu@edu.ucv.ro (C.I.S.); 2 Doctoral School of Chemical Engineering and Biotechnology, National University of Science and Technology POLITEHNICA Bucharest, 1–7 Gheorghe Polizu St., Sector 1, 011061 Bucharest, Romania; oana.micurda@stud.chimie.upb.ro; 3 Faculty of Medicine, University of Medicine and Pharmacy, Petru Rares, 2, 200349 Craiova, Romania; bogdan.oprea@umfcv.ro

**Keywords:** KEDG peptide, new corrosion inhibitor, adsorption properties, experimental study, DFT calculation, Monte Carlo simulation

## Abstract

Experimental and theoretical studies were applied to investigate the adsorption properties of testagen (KEDG) peptide on copper surfaces in sodium chloride solution and, implicitly, its inhibition efficiency (IE) on metal corrosion. The tetrapeptide synthesized from the amino acids lysine (Lys), glutamic acid (Glu), aspartic acid (Asp), and glycine (Gly), named as H-Lys-Glu-Asp-Gly-OH, achieved an inhibition efficiency of around 86% calculated from electrochemical measurements, making KEDG a promising new copper corrosion inhibitor. The experimental data were best fitted to the Freundlich adsorption isotherm. The standard free energy of adsorption ($\Delta G_{ads}^{o}$) reached the value of −30.86 kJ mol^−1^, which revealed a mixed action mechanism of tetrapeptide, namely, chemical and physical spontaneous adsorption. The copper surface characterization was performed using optical microscopy and SEM/EDS analysis. In the KEDG presence, post-corrosion, SEM images showed a network surface morphology including microdeposits with an acicular appearance, and EDS analysis highlighted an upper surface layer consisting of KEDG, sodium chloride, and copper corrosion compounds. The computational study based on DFT and Monte Carlo simulation confirmed the experimental results and concluded that the spontaneous adsorption equilibrium establishment was the consequence of the contribution of noncovalent (electrostatic, van der Waals) interactions and covalent bonds.

## 1. Introduction

Corrosion inhibitors were successfully used to reduce the negative impact of aggressive media on metallic materials, acting by adsorption on their surface leading to the formation of coherently organized protective layers, which interpose at the metal/environment interface, thus delaying the corrosion processes [1]. The inhibition efficiency depends on the compound’s affinity for the metal surface, which determines its mechanism of physical or chemical adsorption. Accordingly, the chemisorption consists of the formation of chemical bonds between some heteroatoms such as oxygen, sulfur, nitrogen, and phosphorus, possessing lone electron pairs, and the vacant orbitals of the atoms from the metal network and/or cations in the electrical double layer, generating specific complexes [1]. Often, chemical adsorption is accompanied by physical adsorption that occurs through weak bonds such as van der Waals forces, hydrogen or hydrophobic bonds, and electrostatic interactions [2].

The combined mechanism of physical and chemical adsorption can be characteristic of amino acids that contain in their molecules both the amino group (–NH_2_) and the carboxylic group (–COOH) and, moreover, a part of them owns the sulfur atom bonded in the thiol side chain (S–H), such as cysteine and acetylcysteine, or S–methyl thioether as in the methionine molecule, as well as the disulfide bond from cystine (the cysteine-oxidized derivative). The amino acids have beneficial properties, being relatively inexpensive, water soluble, without toxicity, and biodegradable [3,4,5]; they do not induce repercussions on human health or the environmental purity, and consequently, they can be successfully used as environmentally friendly corrosion inhibitors [6,7,8,9].

A high inhibition efficiency of 95% of oleic imidazoline and l-cysteine (Cys) on carbon steel corrosion was obtained in CO_2_–saturated NaCl solution at 60 °C for a ratio of 3/1 OIM/CYS [10]. Good performance on mild steel corrosion inhibition in hydrochloric acid using cysteine and its mixture with D–penicillamine (PA) was also communicated in previous studies [11,12].

Phenylalanine’s and aspartic acid’s inhibitory and adsorption properties on mild steel corrosion in HCl solution were investigated by Oubaaqa, M. et al. [13] using electrochemical measurements associated with theoretical techniques such as Density Functional Theory (DFT), Monte Carlo simulation, and molecular dynamics simulation (MDS), concluding that amino acids behave as mixed–type inhibitors and that phenylalanine is relatively less efficient than aspartic acid.

M. Radovanović et al. [14] determined the inhibition performance of L-lysine and L-threonine on stainless steel corrosion in a simulated body solution containing NaCl, KCl, MgCl_2_·6H_2_O, CaCl_2_·2H_2_O, NaH_2_PO_4_·H_2_O, and NaHCO_3_ using potentiodynamic polarization, electrochemical impedance spectroscopy (EIS), and characterization surface techniques such as atomic force microscopy (AFM) and quantum chemical calculation. To improve the adsorption process of the two amino acids, the SS electrode was pretreated in sodium dodecyl sulfate (SDS), obtaining an inhibition efficiency increase up to around 94%.

A wide range of amino acids such as tryptophan, tyrosine, serine, asparagine (Asn), glutamine (Gln), arginine (Arg), serine (Ser), methionine (Met), and valine (Val) were also reported as good corrosion inhibitors for metals/alloys in different media [15,16,17,18,19].

Associated experimental and theoretical studies were implemented that reflected the inhibition ability of amino acids on metal corrosion [16,17,18,20].

Due to the good adsorption properties of amino acids on metal surfaces, their derivatives obtained by intermolecular condensation reactions, such as peptides, were investigated and proposed as corrosion inhibitors for metals/alloys in various media.

The molecular chain of peptides contains multiple active centers capable of attaching on the metallic surface, simultaneously deactivating several vulnerable sites of it and developing protective films that change both the architecture and the characteristics of the surface, thus inducing greater corrosion resistance. Therefore, to reduce the impact of the physiological serum on stainless steel (SS) bioimplants in order to prevent their degradation upon contact with the tissues, the interface architecture was modeled by assembling ecofriendly thin films constituted of hydrolyzed keratin peptides [21].

Small peptides such as L-alanine dipeptide and tripeptide were proposed as potential corrosion inhibitors for iron, copper, and aluminum based on the interpretation of the results taken from computational studies such as Density Functional Theory (DFT) and Monte Carlo simulation (MC) [22]. The inhibitory effect of dipeptides of glycine (Gly) and alanine (Ala) was evaluated by quantum-chemical calculations and Monte Carlo simulation [23]. Also, the experimental and theoretical studies applied to the dipeptide constituted by glycine–glutamic acid (Gly–Glu) and glutathione (a tripeptide of glycine, cysteine, and glutamic acid) revealed their potential as effective inhibitors of carbon steel corrosion [24].

Also, some proteins such as bovine serum albumin [25], bovine corn gluten powder [26], and polydopamine (a synthetic polymer modeled by adhesive proteins from mussels), encapsulating Uio66 loaded with 2-mercaptobenzimidazole [27], were investigated as corrosion inhibitors for different alloys.

The testagen known as KEDG is a tetrapeptide consisting of lysine (Lys), glutamic acid (Glu), aspartic acid (Asp), and glycine (Gly), further presented as H–Lys–Glu–Asp–Gly–OH. KEDG represents a bioregulator intended to stimulate and regulate the functions of the endocrine system. The testagen has the potential to induce the differentiation of stem cells into immune system cells, thus identifying its positive implications for immune function [28,29]. From the point of view of molecular structure, testagen presents the characteristics of a potential corrosion inhibitor. This premise is supported by the fact that lysine and aspartic acid were reported as compounds with adsorption properties, and the inhibitory ability of the dipeptide formed by glycine and glutamic acid (Gly–Glu) was already studied.

Due to its mechanical and electrical properties, copper is used for the manufacture of devices involving heat transfer (cables, pipes, equipment), coils, and electromagnets [30].

Also, its antibacterial properties [31] have recommended it for the production of household utensils, successfully replacing stainless steel, as well as for medical and sanitary applications.

The contact of industrial machinery/equipment manufactured from copper with corrosive environments containing chloride ions induces the metallic oxidation and, implicitly, the occurrence of corrosion processes. Therefore, an unstable and discontinuous layer constituting corrosion products can be formed that affects the surface characteristics and favors the transfer of copper ions from the interface into the contact medium, thus leading to its pollution with unsuitable compounds. Thus, it is necessary to study copper corrosion in a simulated saline environment (0.9% NaCl solution) as well as to implement protection methods that delay its oxidation, respectively corrosion.

Copper is a beneficial metal for the human body, contributing to the health of tissues and blood vessels. However, excess copper caused by its additional ingestion can damage the liver and cause heart and kidney failure, while copper deficiency can impact the number of white blood cells, the heartbeat, or the thyroid. Accidental administration of supplements can create, in one way or another, an imbalance of the metal in the body. Thus, as a preventive measure, it is necessary to study the affinity of certain substances for copper. For example, the tripeptide based on glycine–histidine–lysine (Gly–His–Lys) can bind copper, forming a commercial preparation with antioxidant and anti-inflammatory properties known as GHK-copper tripeptide. Therefore, KEDG that contains both glycine and lysine in its molecule can attach to copper ions, causing its depletion in the body. In this context, the study of the KEDG adsorption properties on copper can signify a motivation from the perspective of anti-corrosion protection as well as a suggestion regarding the risks generated by the random administration, without a medical prescription, of pharmaceutical preparations that can trigger copper imbalance.

The aim of the current study is to investigate a new copper corrosion inhibitor, namely KEDG, in sodium chloride solution using the electrochemical methods associated with SEM/EDS analysis, DFT calculations, and Monte Carlo simulation. KEDG computational study constitutes a new approach to the interactions of a tetrapeptide with the copper surface considering that most studies have been focused on the chemical quantum calculations regarding the adsorption properties of di/tripeptides on different metal surfaces.

## 2. Results and Discussion

### 2.1. Electrochemical Measurements

#### 2.1.1. Open Circuit Potential (OCP) Measurements

**Figure 1:**
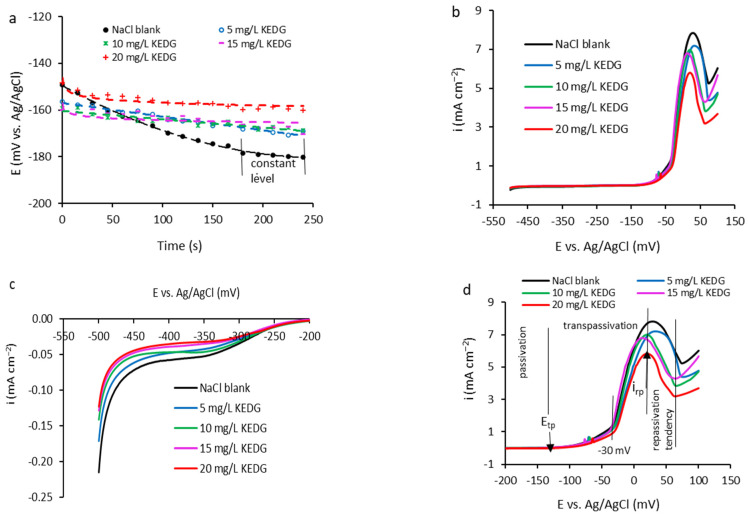
The results of electrochemical measurements: (**a**) OCP measurements; (**b**) the potentiodynamic polarization curves recorded on copper in 0.9% NaCl solution without and with different concentrations of KEDG; (**c**) detail of the cathodic domain; (**d**) detail of the anodic domain.

The OCP measurements applied to the copper specimen surface in 0.9% NaCl solution without and with KEDG, for a time of 4 min (Figure 1a), designed five curves reflecting different metal behaviors depending on the inhibitor concentration.

Generally, metals with relatively high corrosion resistance and a tendency to spontaneous passivation develop a non-uniform upper surface layer disturbed by randomly distributed micro-fissures that can become increasingly profound over time.

Thus, additional active zones can form on which corrosion processes are stimulated, and subsequent measurements can be altered. Consequently, keeping the samples in open circuit for a long time is not recommended. Thus, the copper samples were immersed until primary stabilization of OCP values for a time of 4 min.

In the absence of KEDG, the cyclic adsorption–desorption of NaCl leads to an instability in the upper surface layer and, consequently, larger fluctuations in OCP values. Also, the diffusion rate of the chemical species at the metal surface is significant, affecting strongly the balance in the electric double layer. This leads to Cl^−^ concentration randomly increasing at the copper/electrolyte interface simultaneously with the occurrence of a disturbance in their adsorption–desorption process on the copper surface. Therefore, an irregular surface architecture is configured, leading to a considerable decrease in potential over time. After 165 s, the potential variation is very small, reaching an approximately constant level around −179.8 mV, and after 4 min, an OCP value of −180.3 mV was recorded (Figure 1a—black curve).

In the KEDG presence, the OCP curves shift in the positive direction as the inhibitor concentration increases, revealing a faster stabilization compared with the blank solution. After an immersion time of 4 min, the OCP value increases from −180.3 mV (blank solution) to −159.9 mV at KEDG concentrations of 20 mg L^−1^ (Figure 1a and Table 1). This indicates that KEDG predominantly inhibits the anodic process and the copper oxidation, respectively.

Therefore, the attachment of the KEDG molecular chain to the copper surface by spontaneous physical/chemical adsorption is possible as well as the generation of some complexes between copper ions and the inhibitor, such as [Cu (II)–KEDG], which can be adsorbed onto the substrate through van der Waals forces or other bonds.

Consequently, a protective coating was formed on the copper surface that stabilized the metal/electrolyte interface, leading to the recording of a constant OCP value.

#### 2.1.2. Potentiodynamic Polarization Curves

Figure 1b displays the potentiodynamic curves, i vs. E, in a large potential range of −550 mV and 150 mV. In order to accurately comment on the copper corrosion inhibition phenomenology using KEDG in NaCl solution, the cathodic and anodic polarization curves, respectively, are separately detailed in Figure 1c,d. Investigating the cathodic domain (Figure 1c), it is observed that, at potentials higher than −425 mV, the polarization curves display a plateau at current densities close to zero and over an increasingly extended potential range as the KEDG concentration increases. The experimental data obtained from OCP measurements and unprocessed potentiodynamic polarization curves are presented in Table 1. The standard deviation (SD) is also given.

Thus, in the presence of KEDG, the evolution of the cathodic processes decreases compared with those that take place in its absence, these being considerably limited in NaCl solutions containing 15 mg L^−1^ and 20 mg L^−1^, respectively. In this context, the medium pH being weakly acidic (pH = 5.5), the main cathodic reactions consist of the dissolved oxygen reduction and the hydrogen evolution by the discharge of water molecules simultaneously with the depolarization of hydronium ions (H_3_O)^+^ as a secondary process.

This behavior is associated with a copper spontaneous passivation tendency, which is more pronounced in NaCl solutions containing 15 mg L^−1^ and 20 mg L^−1^, respectively, when the current density approaches zero very closely. At potentials higher than −275 mV, the polarization curves overlap, signaling the transition to the anodic domain, in which distinct areas of copper passivation/activation are highlighted.

The anodic polarization curves (Figure 1d) reveal three distinct zones in the investigated potential range: (1) At the passive domain, located from −200 mV to the transpassivation potential (E_tp_) around −120 mV, the current density maintains at a constant value, practically to zero, and the transfer of ions from the interface into the electrolyte and vice versa is restricted. (2) The transpassive domain manifests itself at potentials higher than E_tp_ (Figure 1d) through the proportional current density increase with the rise in potential, and the copper becomes active, the oxidation processes being stimulated, respectively corrosion (Figure 1d). Between −120 mV and −30 mV, the transpassivation field is characterized by a reduced copper oxidation activity when the current density slightly increases with potential increase; at potentials higher than −30 mV, up to 20 mV, the copper transpassivation is more pronounced, the metal activity becoming intense and highlighted by the steeper increase in current density, reaching a maximum value corresponding to the repassivation current (i_rp_), which decreases with the increase in KEDG concentration (Figure 1d and Table 1). (3) At potentials higher than 20 mV, a very weak tendency of copper repassivation is reconfigured, when the current density slowly decreases from i_rp_ to lower densities, but it does not reach a constant value close to zero over a certain potential range. Copper corroded in blank NaCl solution reactivates at high current densities, and therefore, its repassivation tendency is lower compared with that corroded in NaCl solution containing KEDG.

Analyzing the results from Table 1, it can be observed that (i) the OCP shifts toward higher values with an increasing inhibitor concentration, and the standard deviation decreases, suggesting a better stability of the copper/electrolyte system in the presence of KEDG; (ii) repassivation current (i_rp_) decreases as the KEDG concentration increases, indicating that the inhibitor presence favors copper repassivation and the delay of subsequent corrosion processes, respectively.

All of the above indicates that KEDG acts as an inhibitor of copper corrosion in the NaCl environment, ensuring its protection by forming an adsorbed upper layer on the metal surface, which restricts oxidation processes and charge transfer between metal and electrolyte.

#### 2.1.3. Tafel Polarization and Linear Diagram

**Figure 2:**
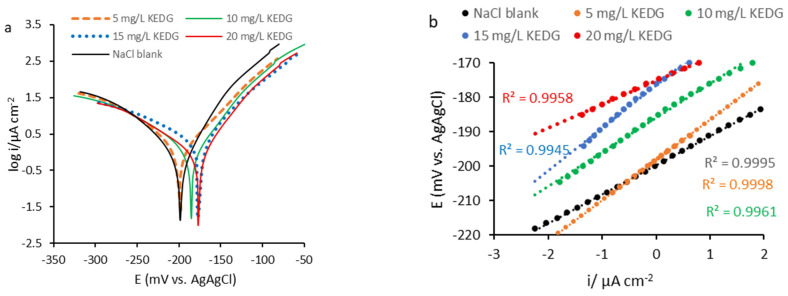
Processed potentiodynamic polarization curves for copper corroded in NaCl blank solution and NaCl solution containing various KEDG concentrations: (**a**) semilogarithmic curves (Tafel polarization); (**b**) linear diagram recorded in overvoltage range of ±10 mV in respect to the corrosion potential.

As shown in Figure 2a, in the presence of KEDG, the corrosion potential (E_corr_) slightly shifts in the positive direction, and the semilogarithmic polarization curves follow the same trend simultaneously with their displacement in the lower current areas. This behavior is associated with the decrease in corrosion current density, which is more pronounced in the NaCl solution containing 20 mg L^−1^ KEDG. As can be seen in Figure 2a, the maximum displacement of E_corr_ is less than 85 mV, suggesting that KEDG acts as a mixed inhibitor [32], the anodic process being affected to a greater extent compared with the cathodic one. Therefore, KEDG is a predominantly anodic inhibitor, acting by adsorption on the copper surface and forming an organic film that blocks the active sites on the metal surface.

According to electrochemical kinetics [2,21], the anodic and cathodic Tafel equations can be applied to potential values far from the corrosion potential. Thus, the following conditions are imposed: η ≥ 52 mV and E >> E_corr_ for anodic process and η ≤ −52 mV and E << E_corr_ for cathodic ones, respectively.

Therefore, the corrosion current was determined at the intersection of the Tafel lines extrapolated to the corrosion potential, according to the anodic and cathodic Tafel equations (Equations (1) and (2)).

(1) $$\eta =b_{a}logi-b_{a}\log i_{corr}$$

(2) $$\eta =b_{c}logi_{corr}-b_{c}\log i$$

where i—current density; i_corr_—corrosion current density; η—overvoltage; b_a_ and b_c_—anodic and cathodic Tafel slopes expressed by Equations (3) and (4):

(3) $$b_{a}=({\frac{d\eta }{dlogi})}_{\eta \gg 52mV}=\frac{RT}{zF\alpha }$$

(4) $$b_{c}=(-{\frac{d\eta }{dlogi})}_{\eta \leq -52mV}=-\frac{RT}{zF\beta }$$

where z—number of exchangeable electrons in the investigated system; α and β—kinetic transfer coefficients; α + β =1; F—Faraday’s constant = 96,500 C mol^−1^; R—the universal constant of gases = 8.31 J mol^−1^ K^−1^; T—temperature (K).

Figure 2b shows the voltage–current polarization curves (E vs. i) recorded in potential zones very close to the corrosion potential and whose linearization was performed in the overvoltage range = ±10 mV.

The polarization resistance (R_p_) represents the slope of the lines designed in Figure 2b, for potential values close to E_corr_ (Equation (5)), and it simultaneously satisfies the Stern–Geary equation (Equations (6) and (7)), respectively.

(5) $$R_{p}=({\frac{dE}{di})}_{E\to E_{corr}}$$

(6) $$R_{p}(\Omega \;{cm}^{2})=\frac{b_{a}xb_{c}}{2.303(b_{a}+b_{c})}\times \frac{1}{i_{corr}}$$

(7) $$R_{p}(\Omega \;{cm}^{2})=\frac{RT}{zF}\times \frac{1}{i_{corr}}$$

Based on the theoretical considerations shown above, electrochemical parameters such as E_corr_, i_corr_, R_p_, and b_a_ and b_c_ were calculated using VoltaMaster 4 software.

The inhibition efficiency of KEDG on copper corrosion in NaCl solution was determined according to the values of i_corr_ and R_p_ recorded after potentiodynamic polarization in the absence of KEDG ($i_{corr}^{o}\;and\;R_{p}^{o},\;respectively$) and in its presence (i_corr_ and R_p_, respectively), using Equations (8) and (9) [32,33,34,35]. All acquired experimental data are presented in Table 2.

(8) $$IE=\frac{i_{corr}^{o}-i_{corr}}{i_{corr}^{o}}\times 100$$

(9) $$IE=\frac{R_{p}-R_{p}^{o}}{R_{p}}\times 100$$

#### 2.1.4. Potentiogravimetric Results

The potentiodynamic curves were processed as semilogarithmic polarization curves that were used to compute, with VoltaMaster 4 software, the corrosion rate (CR) using the potentiogravimetric method.

The conversion of the corrosion current density (i_corr_) into corrosion rate, expressed in μm Year^−1^, is revealed by Equation (10) [21].

(10) $$CR(\mu m\;{Year}^{-1})=\frac{i_{corr}A}{1000zF\rho }\times 24\times 365\times 10^{6}$$

where z is the number of electrons interchangeable in the process; i_corr_ is the corrosion current density (A m^−2^); A represents copper atomic mass (g mol^−1^); F is Faraday’s constant (26.8 A h mol^−1^); ρ is copper density (kg m^−3^); 24 and 365 are multiplication factors for hours and days, respectively; 1000 and 10^6^ are the correction factors for the density unit from Kg m^−3^ in g m^−3^ and the transformation factor for CR unit from meters (m) to μm, respectively. The potentiogravimetric results are also presented in Table 2.

The inhibition efficiency was calculated using Equation (11), and the surface coverage degree (θ) with adsorbed inhibitor molecules was determined as IE/100.

(11) $$IE=\frac{{CR}^{o}-CR}{{CR}^{o}}\times 100$$

where CR^o^ is the copper corrosion rate in the absence of the KEDG inhibitor and CR is the copper corrosion rate in the presence of KEDG.

Inspecting the data from Table 2, it is observed that i_corr_ and CR decrease while R_p_, IE, and θ increase with increasing inhibitor concentration. Thus, IE value of around 86% was obtained at a KEDG concentration of 20 mg L^−1^.

The Tafel slopes (b_a_ and b_c_) show small variations for the copper corrosion in the inhibited solutions, registering a slight decrease compared with those obtained for the metal oxidation in the uninhibited solution, which suggests that the mechanism of the corrosion process remains unchanged. The decrease in b_a_ values suggests that, when the inhibitor film is adsorbed, a blocking effect on surface active sites takes place [36]. The slight variation in b_c_ indicates a low action of KEDG on the evolution of cathodic reactions.

Literature data survey: Certain peptides have been researched as corrosion inhibitors for copper, mild steel, and 304L stainless steel in acidic and saline environments using various experimental methods associated with surface characterization techniques and computational studies. Zhang, Da-Quan et al., 2009 [37] investigated glutathione (Glt), a tripeptide constituted from glycine, cysteine, and glutamic acid as copper corrosion inhibitor by the mass-loss technique and electrochemical measurements establishing maximum inhibition efficiency of about 92.7%, for 10 mmol L^−1^ Glt concentration in 0.5 mol L^−1^ HCl solution. Andjela Simović, A. et al., 2023 [24] studied Glt as a corrosion inhibitor for mild steel, determining a high inhibition efficiency of this tripeptide of 97.3% in 1.0 mol L^−1^ HCl solution. Samide, A. et al., 2024 [21] reported a mixture of hydrolyzed keratin peptides (HKER) synthesized from sheep wool by alkaline hydrolysis as a 304L stainless steel corrosion inhibitor in physiological serum (0.9% NaCl solution) using electrochemical measurements associated with optical microscopy and atomic force microscopy (AFM). The corrosion tests were accomplished for various concentrations of HKER and at four temperatures, revealing that the inhibition efficiency reaches the maximum value around 88%, at 25 °C and 40 mg L^−1^ HKER, in physiological serum.

### 2.2. Approach of Adsorption Isotherms

The best way to quantitatively express the binding affinity of organic compounds on metal surfaces is provided by adsorption isotherms that allow the calculation of adsorption–desorption equilibrium constants (K) accessible to the subsequent evaluation of the standard free energy of adsorption ($\Delta G_{ads}^{o}$), constituting an opportunity for the adsorption type identification (physical or chemical).

Consequently, in order to evaluate the KEDG adsorption on the copper surface, six adsorption isotherms were verified, the linearized form of each of them being displayed in Table 3. The surface coverage degree values were taken from the potentiogravimetric measurements that are displayed in Table 2.

**Figure 3:**
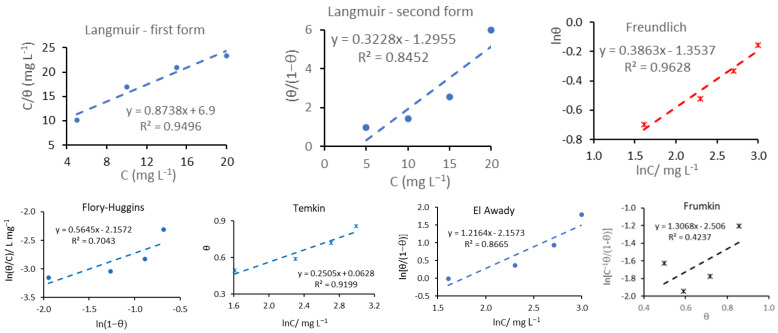
Graphical representation of the applied models for the adsorption process of the KEDG on the copper surface.

The results obtained by designing the linearization functions (Table 3) are illustrated in Figure 3. The R^2^ coefficient expressing the deviation from linearity (squared value on chart), as well as the equations of the drawn lines, are inserted in each graph, these being used to determine the slope and the intercept with the ordinate and therefore K, respectively (listed in Table 3).

Analyzing the values of R^2^ coefficients inserted in Table 3 and Figure 3, it can be seen that the Freundlich isotherm represents the most suitable model for KEDG adsorption on the copper surface, in this case the fitting of the experimental data being achieved with the highest R^2^ coefficient (0.9628).

It can be observed that for Langmuir, Flory-Huggins, Temkin, and El-Awady’s adsorption models, lower linearity coefficients (R^2^) were obtained compared with the one resulting from the Freundlich isotherm, and Frumkin is not applicable. The experimental data obeyed the first linearized form of the Langmuir isotherm with R^2^ close to 0.95, but with a slope of 0.8738. Consequently, if the slope deviates from unity (Table 3), the applicability of the Langmuir adsorption isotherm is limited. Moreover, different values for K were obtained from the two Langmuir linearized equations, which also induces reluctance in its application for KEDG adsorption on the copper surface.

In order to determine the standard free energy of adsorption ($\Delta G_{ads}^{o}$) using Equation (14) [38], the adsorption–desorption equilibrium constant (K) was calculated from the Freundlich isotherm based on Equations (12) and (13).

Freundlich parameters (K, n) include all the factors that influence the adsorption process, and these can be calculated from the logarithmic representation of the Freundlich equation, lnθ = f(lnC). Thus, the slope of the line corresponding to the Freundlich isotherm (Figure 3) is equal to 1/n, and the intersection with the ordinate represents lnK.

(12) lnK=−1.3537

(13) $$K=e^{-1.3537}$$

(14) $$\Delta G_{ads}^{o}=-RTln(10^{6}K)$$

where R is the universal constant of gases (8.31 J mol^−1^ K^−1^); T is the thermodynamic temperature (K); and 10^6^ is the water concentration expressed in mg L^−1^ as a result of the fact that KEDG concentration was expressed in mg L^−1^, and consequently, the adsorption–desorption equilibrium constant (K) was calculated in L mg^−1^.

Freundlich adsorption isotherm application limit: In this case, the equation inserted in the graph from Figure 3 (Freundlich) can be applied up to a maximum KEDG concentration of 33.15 mg L^−1^. At KEDG concentrations higher than 33.15 mg L^−1^, θ exceeds the unity, and consequently, the Freundlich isotherm is unreliable.

Some clarifications are needed, as follows: (i) the Freundlich isotherm describes non-ideal and reversible inhibitor adsorption on heterogeneous surfaces; (ii) the vulnerable active centers of the metal surface are preferentially occupied by the inhibitor molecular chain; (iii) the adsorption is not restricted by the preabsorbed surface layer formed by other molecules from the electrolyte [42,43].

Consequently, KEDG is suitable for occupying high-activity surface sites located around impurities or metal network defects. KEDG adsorption is not blocked by the passive layer formed on the copper surface. The inhibitor can be embedded in the oxide layer, improving the protective coating adhesion on the copper surface.

Moreover, depending on the value of the Freundlich factor (n), the adsorption can be classified into (a) unfavorable, if n < 1, respectively 1/n > 1; (b) favorable, if n > 1, and 0 < 1/n < 1, respectively; (c) the adsorption is irreversible if “n” reaches a very high value; in this case, 1/n approaches zero.

The values of the Freundlich parameters and the adsorption standard free energy ($\Delta G_{ads}^{o}$) are shown in Table 4.

Consequently, the KEDG inhibitor has a good affinity for copper, its adsorption on the surface being favored (n = 2.58) by certain KEDG–copper interactions that may involve both chemical and physical bonds. As Table 4 shows, a negative value was obtained for ${\Delta G}_{ads}^{o}$, indicating the KEDG spontaneous adsorption on the copper surface. As is known, the limit-threshold of the chemical adsorption is −40 kJ mol^−1^ [44]. For the KEDG adsorption, the ${\Delta G}_{ads}^{o}$ value of −30.86 kJ mol^−1^ > −40 kJ mol^−1^ was obtained, indicating that the KEDG inhibitor acts through a mixed mechanism, in which physical adsorption prevails over the chemical one. Therefore, KEDG binding on the copper surface is favorable and spontaneous, involving a relatively strong physical adsorption process accompanied by a chemically moderate one.

Exploration of the literature data:

Similar results have been reported for ${\Delta G}_{ads}^{o}$ values regarding the adsorption of other peptides investigated as corrosion inhibitors for certain metallic materials in different aggressive environments.

Thus, for the glutathione tripeptide adsorption on the copper surface in 0.5 mol L^−1^ HCl solution, the Langmuir isotherm was identified, with a ${\Delta G}_{ads}^{o}$ value around −32 kJ mol L^−1^, indicating a strong physical adsorption [37].

The adsorption process of a mixture of hydrolyzed keratin peptides (HKER) on a 304L stainless steel surface in physiological serum was carried out according to the El-Awady model, finally obtaining ${\Delta G}_{ads}^{o}$ values of −26.5 kJ mol^−1^ at 25 °C and −28.7 kJ mol^−1^ at 55 °C. This behavior characterizes the prevalence of physical adsorption over the chemical one [21].

The inhibition action of the extract of albumin (a family member of globular proteins obtained from egg white) on low-carbon steel corrosion was tested in 1 N HCl solution and 1 N H_2_SO_4_, respectively. The best fitting of the experimental data was realized in agreement with the Langmuir adsorption isotherm. In this case, ${\Delta G}_{ads}^{o}$ for the albumin adsorption on the low-carbon steel surface reached higher negative values than −20 kJ mol^−1^, thus revealing a predominantly spontaneous physisorption process [45].

### 2.3. Surface Characterization

The changes in the morphology of the copper surface corroded in NaCl solution, in the absence and presence of KEDG, compared with an uncorroded control sample (standard), were identified from images acquired by optical microscopy and scanning electron microscopy (SEM) assisted by energy-dispersive X-ray spectroscopy (EDS).

#### 2.3.1. Optical Microscopy

**Figure 4:**
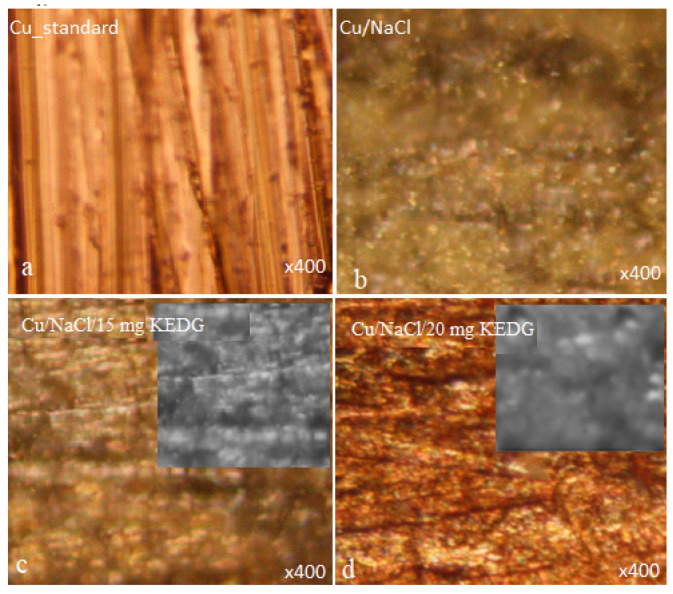
Optical microscopy images acquired for copper samples: (**a**) standard copper before corrosion; (**b**) copper corroded in NaCl blank solution; (**c**) copper corroded in NaCl solution containing 15 mg L^−1^ KEDG; (**d**) copper corroded in NaCl solution containing 20 mg L^−1^ KEDG.

Figure 4 displays the optical microscopy images acquired before (Figure 4a) and after the copper corrosion in 0.9% NaCl solution, without (Figure 4b) and with the tested inhibitor (Figure 4c,d).

The standard (Figure 4a) presents a specific morphology of the uncorroded copper surface. After corrosion in NaCl without KEDG (Figure 4b), significant morphological changes can be noticed caused by the copper oxidation followed by the random adsorption of corrosion products (e.g., CuO) on the metal surface. Thus, on the copper surface, a non-uniform layer develops, disturbed by some micro-zones, on which the corrosion processes can be reactivated.

In the presence of KEDG, the images in Figure 4c,d show a relatively more uniform layer, with an apparently different texture than that revealed in the KEDG absence, having a slightly globular profile, as shown in the inserted details.

#### 2.3.2. SEM/EDS Analysis

**Figure 5:**
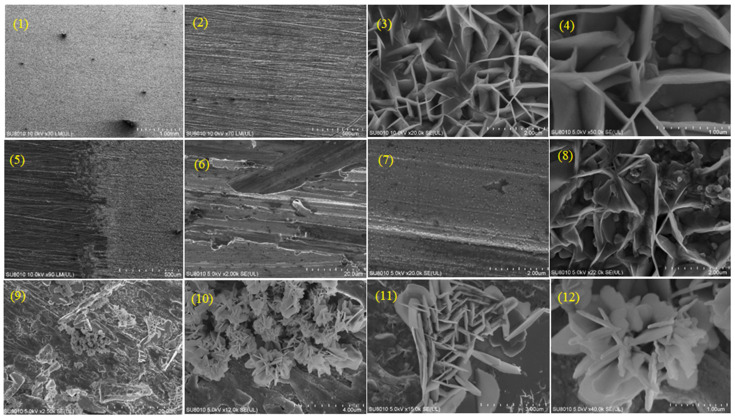
SEM images collected at different resolutions and magnifications before and after copper corrosion in 0.9% NaCl solution, in the absence and presence of KEDG: copper sample before corrosion (standard)—slides (**1**–**4**); threshold between uncorroded and corroded copper surface—slide (**5**); copper corroded in NaCl solution without KEDG—slides (**6**–**8**); copper corroded in NaCl solution containing KEDG—slides (**8**–**12**).

As shown in Figure 5, in order to distinguish the morphological differences/similarities of the copper surface before and after corrosion in 0.9% NaCl solution, with and without the tested inhibitor, SEM images were acquired for several resolutions associated with distinct magnifications, such that fine details could be accurately highlighted.

The resolution and magnification are fundamental concepts in SEM, constituting the main elements in the image formation and interpretation. Resolution shows the ability to visualize tiny features, while magnification refers to the enlarging of the sample view.

Images from (1) to (4) show the copper surface before corrosion (control sample). Slides (1) and (2) (resolutions: 1 mm and 500 μm, respectively) show a generally homogeneous copper surface typical of a sample undisturbed by the attack of the external environmental factors. Certain defects are evidenced, more or less originating from the metal processing, or from the mechanical impact of handling on the copper foil. The surface details revealed at higher magnifications (images (3) and (4)), in which a coherent metallic network distorted in places by ineluctable imperfections can be observed.

To observe the morphological/textural differences between the standard and corroded samples, slide (5) shows the areas located in the proximity of the demarcation threshold between the copper plate surface immersed in NaCl solution (post-corrosion, right) and the one remaining in contact with the atmosphere (pre-corrosion, left). The uncorroded surface provides similar characteristics to the control sample (slide 2) compared with the one corroded in NaCl solution without KEDG, displaying a changed morphology, which no longer highlights the same specific particularities of those of the standard. Images (6), with 20 μm resolution, and (7), with 2 μm resolution, show the development of an ambiguous upper surface layer that is difficult to evaluate visually, indicating changes in the morphology of the copper surface corroded in NaCl solution. Some surface defects are less noticeable, the investigated sample being covered with a layer disturbed by some microdeposits, most likely consisting of saline formations (NaCl). This is more pronounced in image (8), having a 2 μm resolution and higher magnification, which shows a surface with multiple changes compared with the standard sample (slide 3), profiling itself a network completely distorted by the saline solution’s corrosive attack on the copper surface.

In the presence of KEDG, the surface morphology highlights the occurrence of more or less pronounced (image 9) microdeposits of needle-shaped blooms’ appearance (image 10). These seem to be distributed in a network similar to a fabric (slide 11) entered into a matrix surrounded by regular borders that give a flower-like appearance (slides 11 and 12).

**Figure 6:**
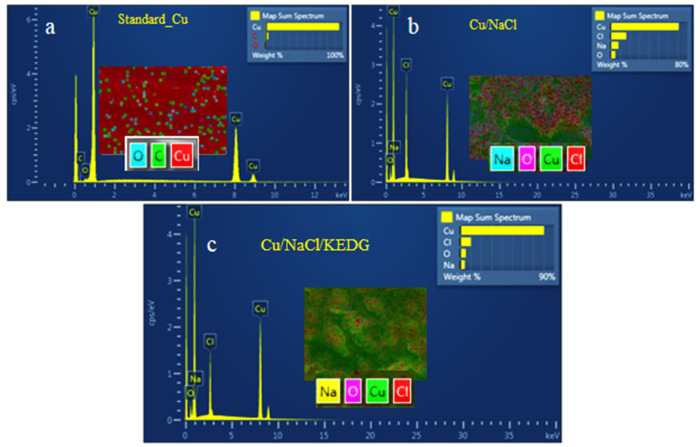
EDS spectra recorded for (**a**) control copper sample before corrosion; (**b**) copper corroded in NaCl solution without KEDG; (**c**) copper corroded in NaCl solution containing KEDG.

This morphological characteristic can be associated with an adsorbed layer on a metallic surface consisting of KEDG, NaCl, and copper oxides. The results were confirmed by EDS analysis, displayed in Figure 6.

The analysis of the EDS spectra from Figure 6 can be summarized as follows: (i) The control sample before corrosion (Figure 6a) is a pure, unalloyed metallic copper with very small amounts of oxygen and carbon, with an insignificant impact on the metal composition. (ii) After the corrosion in NaCl solution in the absence of the inhibitor (Figure 6b), sodium and chloride ions originated from the electrolyte, as well as the oxygen from CuO generated by the copper oxidation, were identified, these being randomly scattered on the surface. It is worth noticed that a change in the surface color from copper-reddish to black was evidenced during the experiments, which can be caused by black CuO electrodeposited during the corrosion tests. (iii) In the presence of KEDG (Figure 6c), the amounts of secondary species (Na, Cl, O) decrease compared with those formed in its absence (Figure 6b), followed by the simultaneous increase in copper concentration from 80% to 90%.

This indicates that the KEDG presence leads to the formation of a protective coating interposing at the metal/electrolyte interface, thus blocking/delaying the copper oxidation and its corrosion, respectively.

Note that the nitrogen from the KEDG molecule was not detected in the EDS spectrum. More or less hypothetically, KEDG was masked by the other compounds that form the protective layer and/or nitrogen, being in very low concentration, was below the detection limit. Also, the carbon from the KEDG molecules was absorbed by the carbon baseline.

Based on the above, it can be stated that a composite coating was adsorbed on the copper surface, containing KEDG as well as NaCl and copper corrosion compounds in small concentrations.

### 2.4. Investigation of Interactions at the Copper/KEDG Interface Using the DFT Method

**Figure 7:**
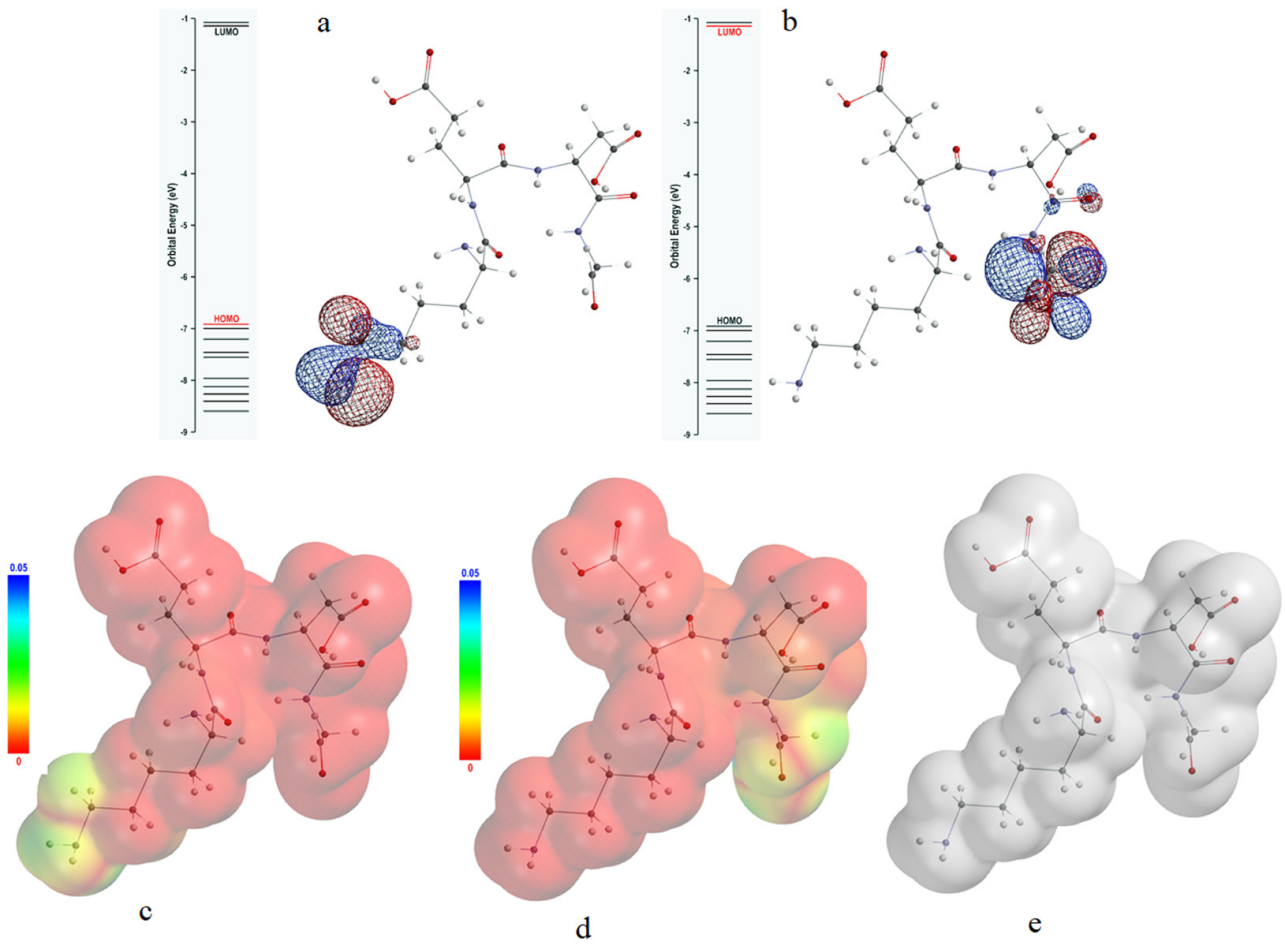
Distribution and mapping of the HOMO (**a**,**c**) and LUMO (**b**,**d**) frontier molecular orbitals of KEDG on the isosurface of the total electric charge density (**e**).

The contact interactions between the Lys-Glu-Asp-Gly (KEDG) and the copper surface are due to the distribution of electron density in the tetrapeptide molecular chain. According to Fukui’s frontier molecular orbital theory, electron transfer interactions are mediated by the HOMO and LUMO molecular orbitals, their energy position determining the donor–acceptor interaction type of KEDG with the copper surface. The distribution analysis of frontier molecular orbitals (Figure 7a,b) was performed, and their magnitudes of energies were evaluated.

In our investigation, a significant part of the HOMO orbital was found to be distributed in the region of the amino group at the end of the lysine molecular chain, and the other surrounds the carbon atoms from its vicinity.

The LUMO orbital proved to be strongly localized in the region of the carboxyl group of the glycine as well as in the region of the peptide bond between the molecular chains originating from aspartic acid and glycine. Therefore, a bidirectional electron flow between the KEDG molecule and the copper surface is possible, the electron donor sites being located at the amine group of the lysine chain, while the electron acceptor sites are placed at the glycine carboxyl group.

The less negative energy of the HOMO orbital and the spatial extension of its main distribution lobes can be favorable to a high overlapping degree, suggesting that it is most likely for the tetrapeptide to behave as an electron donor within donor–acceptor interactions.

The relatively small energy gap between LUMO and HOMO (Equation (15)) of 5.47 eV can be correlated with a good reactivity of the KEDG molecule, thus explaining the small modification of the molecular electron cloud’s easy modification through the mutual exchange of electrons between the KEDG and the copper surface.

(15) $$\Delta E=E_{LUMO}-E_{HOMO}=-1.14-\left(-6.61\right)=5.47eV$$

An estimation of the improvement in the electron transfer direction within donor–acceptor interactions was obtained by the distribution mapping of the frontier molecular orbitals onto the isosurface of the total charge density calculated for the KEDG molecule (Figure 7e). Comparative visual analysis of the frontier molecular orbital isosurfaces (Figure 7c,d) reveals the prevalence of nucleophilic behavior of the KEDG molecule. Therefore, it is most likely for the electron transfer to occur from the KEDG molecule to the Cu (1 1 1) surface.

**Figure 8:**
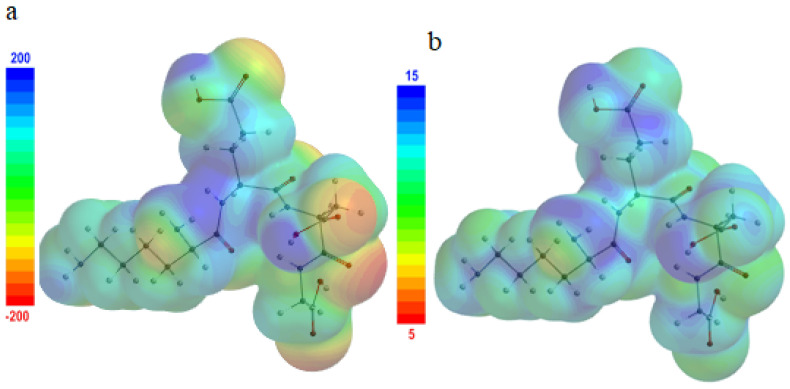
A 3D map of (**a**) the molecular electrostatic potential; (**b**) the local ionization potential.

Additional information regarding the location of electron-rich molecular sites was obtained by mapping the molecular electrostatic potential (Figure 8a) onto the total charge density isosurface (Figure 7e). As the relative color scale indicates, nucleophilic centers associate with the molecular regions with high electron density, corresponding to negative values of the electrostatic potential, coded in red.

At the same time, the 3D map of the electrostatic potential (Figure 8a) reveals the planarity of the testagen molecules, which allows them to orient parallel to the copper surface.

Thus, the number of contact points with the copper surface, and consequently, the surface coverage degree, respectively, are maximized.

The KEDG molecule regions susceptible to easily donate electrons to Cu^2+^ ions from the electric double layer were also identified using the simulated local ionization potential map (Figure 8b), being associated with low values of ionization energy.

**Figure 9:**
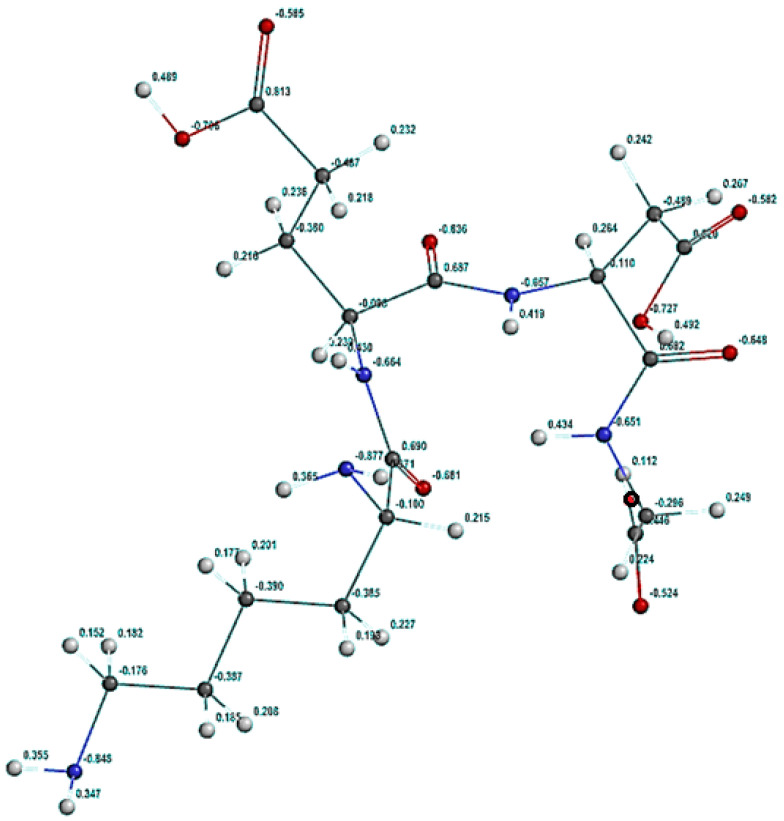
Natural charges calculated using the NBO method. Assigned colors to: dark gray—carbon atoms; light gray—hydrogen atoms; blue—nitrogen atoms; red—oxygen atoms.

The partial atomic charges (Figure 9) were calculated using the Natural Bond Orbital (NBO) method [2,16], which allowed the identification of the atoms with significant negative partial charges, these constituting the KEDG molecule active centers in the adsorption process on the copper surface.

As shown in Figure 9, the significant negative charges are distributed to (1) the nitrogen atoms in the side chain (around −0.900); (2) the simple-linked oxygen atoms attached to the carboxyl group (around −0.700); (3) the oxygen atoms in the peptide group (around −0.650); (4) the nitrogen atoms in the peptide group (around −0.650); (5) finally, the double oxygen attached to the carboxyl group (around −0.550).

Thus, these atoms are the main active centers that can determine the KEDG attachment to the copper surface, facilitating the adsorption process. Also, the extended electron cloud around some carbon atoms, generating their negative charges (between −0.400 and −0.300), can have a secondary role in the KEDG adsorption process on the copper surface.

The magnitude of the negative partial charges indicates the adsorption ability of the KEDG molecules on the copper surface through donor–acceptor and electrostatic interactions.

Reactivity global descriptors

In order to elucidate the nature and strength of interactions at the Cu(111)/testagen interface, reactivity global descriptors were also estimated, the calculation being performed using the CDFT (Conceptual Density Functional Theory) methodology. Based on the literature data, the main descriptors, such as χ (electronegativity), ε (chemical potential), η (hardness), σ (softness), ΔN (the fraction of the transferred electron), and ω (electrophilicity index) were calculated using Equations (16)–(21) [46,47].

(16) $$\chi =\frac{I+A}{2}\approx -\frac{E_{\mathrm{LUMO}}+E_{\mathrm{HOMO}}}{2}$$

(17) ε=−χ

(18) $$\eta =\frac{I-A}{2}\approx \frac{E_{\mathrm{LUMO}}-E_{\mathrm{HOMO}}}{2}$$

(19) $$\sigma =\frac{1}{\eta }$$

(20) $$∆N=\frac{\varphi_{Cu}-\chi_{inh}}{2\left(\eta_{FCu}+\eta_{inh}\right)}$$

(21) $$\omega =\frac{\mu^{2}}{2\eta }=\frac{\chi^{2}}{2\eta }$$

The vertical ionization energy (I) and the vertical electron affinity (A) were approached in terms of the energies of the frontier molecular orbitals, HOMO and LUMO (Equations (22) and (23)).

The calculation was based on the eigenvalues of the virtual Kohn–Sham orthogonalized orbitals.

I = −E_HOMO_ (22)

A = −E_LUMO_ (23)

Table 5 presents the main reactivity global descriptors obtained for KEDG compared with the data reported by Kumar D. et al., 2020 [3] for glutathione and the two amino acids common to peptides, namely, glutamic acid and glycine. The inhibitor adsorption ability depends on the ΔE value, its molecules having a higher affinity for the metallic surface, as the energy gap between the frontier molecular orbitals HOMO and LUMO decreases. As can be seen in Table 5, for KEDG, ΔE reached the lowest value of 5.47 eV, indicating a higher adsorption capacity compared with glutathione, glutamic acid, and glycine, respectively.

This is explained by the increased number of the KEDG molecule active centers, which is constituted by four amino acids (lysine, glutamic acid, aspartic acid, and glycine) compared with glutathione, whose molecule is made up of three amino acids (cysteine, glutamic acid, and glycine).

The peptide molecular group (–CO–NH–) participates in the electron acceptation–donation processes, thus interacting with the copper surface. It also represents an additional adsorption center, which can stimulate the surface/peptide interactions, enhancing the inhibitory effect. Moreover, based on theoretical calculations, it was shown that the inhibition efficiency, respectively, the surface coverage degree, increases with the increase in the length of the peptide molecular chain. At the same time, the organization of long chains during immersion in the corrosive environment results in a compact film structure restricting electrolyte penetration, which was proven by SEM/EDS analysis.

On the one hand, a higher electronegativity (χ) value reflects a higher ability of molecules to accept electrons, indicating that KEDG attracts electrons comparatively with the glutathione molecule (χ values are very close), but less than the individual amino acids. On the other hand, if the hardness value (η) decreases and softness (σ) increases, the inhibitor is more easily adsorbed. Although the KEDG molecule provides several active centers that enhance adsorption ability, its inhibition performance is due to the functional groups –NH_2_ and –COOH and the peptide group (–CO–NH–), which adhere to the surface through weak physical bonds. Due to the –SH group from the cysteine molecule, glutathione can be fixed to the surface through stronger bonds, providing a higher stability to the adsorbed film. However, it does not prevail over the KEDG adsorption ability.

It can be noticed that the differences between the descriptors are very small (especially softness), signifying that both glutathione and KEDG reach a good inhibition performance of the copper corrosion.

The transferred electron fraction (ΔN) is positive, indicating that the transfer of electrons takes place from the KEDG molecules to the copper surface, while the electrophilicity index (ω) is in agreement with the electronegativity (χ), showing that the KEDG represents a weaker electron acceptor than glutathione. Also, larger ΔN values indicate a stronger molecule capability to donate electrons [48], meaning that KEDG is a better electron donor than glutathione.

The analysis of the descriptors presented in Table 5 indicates that the two amino acids (glutamic acid and glycine) provide lower adsorption abilities than peptides (testagen and glutathione).

**Scheme 1:**
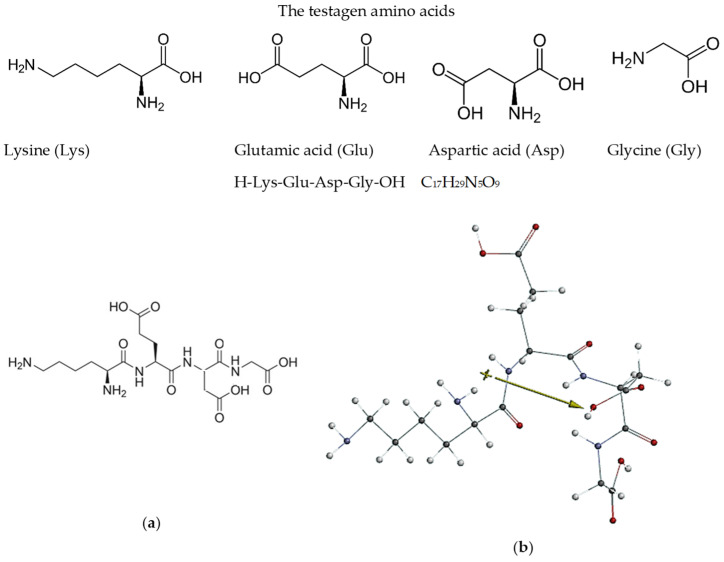
Amino acids constituting KEDG tetrapeptide and KEDG structure: (**a**) KEDG molecular formula; (**b**) molecular geometry in the aqueous phase of KEDG in the singlet ground state and electric dipole orientation.

To support the above discussion, the dipole moment (μ) was computed (Scheme 1b, Section 3.1). The high value of the dipole moment (11.83 D), correlated with the low value of ΔE (5.47 eV), reveals that KEDG exhibits high adsorption ability on the copper surface and provides a good inhibition efficiency, in agreement with the experimentally obtained results.

The correlative analysis of the reactivity global descriptors shows that the KEDG ensures good protection of the copper surface. Also, KEDG participates in the electron-acceptance–donation processes, its nucleophilic character being thermodynamically favored and clearly predominating the electrophilic one.

Brief review of the literature data:

Simović A. et al., 2023 [24] tested six groups of compounds as corrosion inhibitors for mild steel in 1 mol L^−1^ HCl solution, as follows: (1) glycine (Gly); (2) glutamic acid (Glu); (3) cysteine (Cys); (4) a mixture of three amino acids glycine, glutamic acid, and cysteine (Gly + Glu + Cys); (5) a dipeptide mixture composed of glycine and glutamic acid (Gly-Glu) and the amino acid cysteine (Gly-Glu + Cys); (6) a tripeptide composed of glycine, cysteine, and glutamic acid [glutathione (Glt)]. Based on theoretical calculations, it was demonstrated that the inhibition performances of the investigated systems increased with increasing peptide molecular chain length.

Also, Fawzi Nassar M. et al., 2022 [49] showed that the enhancement of the inhibitory effect of di- and tri-peptides increases according to the increase in the number of peptide active centers.

Kasprzhitskii, A. and Lazorenko, G., 2021 [22] evaluated the local and global descriptors of the reactivity of alanine (Ala)-based peptides, namely the dipeptide Ala–Ala and the tripeptide Ala–Ala–Ala. They reported an increase in the inhibitory effect with the increase in the compound’s molecular chain length due to the supplementation of the active centers from the peptide molecular structure. The adsorption ability of small peptides was indicated by the following sequence: (Ala) > (Ala–Ala) > (Ala–Ala–Ala). Also, the peptides ensured a better adsorption capability on the iron surface than on the copper one.

Kasprzhitskii A. et al., 2021 [23] investigated the role of the peptide bond in enhancing the inhibitory effect of the compounds based on aliphatic amino acids such as alanine (Ala) and glycine (Gly) using computational studies. It was found that the peptide group is involved in electron acceptance–donation processes and interacts with the metal surface. The peptide group constitutes an additional active center that can stimulate the interaction and, respectively, adsorption on the metal surface, thus improving the inhibitory effect. According to Monte Carlo simulation results, an aliphatic dipeptide based on Ala and Gly highlighted an improved inhibitory effect compared with individual amino acids.

### 2.5. Atomistic Monte Carlo Simulation

**Figure 10:**
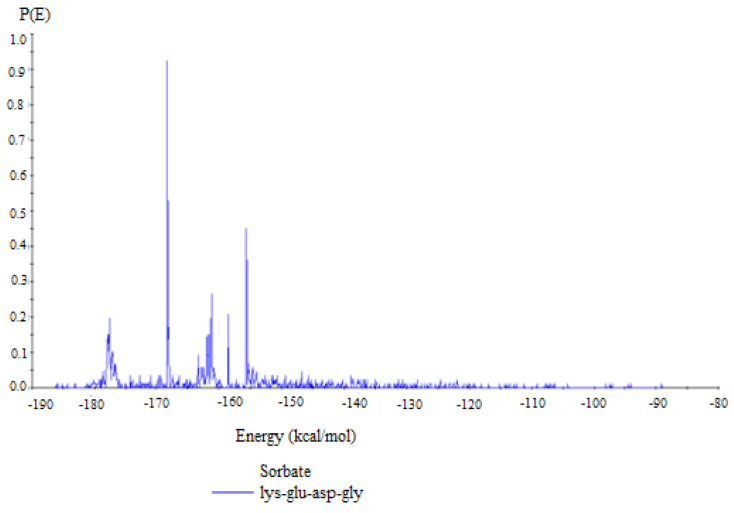
Potential energy distribution profile of KEDG molecules adsorbed on the Cu(1 1 1) surface.

The lowest energy adsorption configuration was identified by exploring the configuration space of the Cu (1 1 1)/Lys-Glu-Asp-Gly system using the simulated annealing method involving locating the global energy minimum through a metaheuristic algorithm. Canonical Monte Carlo sampling of the search space resulted in 196 low-energy adsorption configurations, with the lowest estimated adsorption energy being −186.027 kcal mol^−1^ (−44.46 kJ mol^−1^) and the highest estimated value being −89.200 kcal mol^−1^ (−21.32 kJ mol^−1^). The simulation of adsorption scenarios allowed the derivation of the range of energies for different KEDG configurations at its interaction with the given Cu(1 1 1) surface. The result of compiling into a distribution profile showing the energy values against the corresponding probabilities is shown in Figure 10.

The position of the peaks in the distribution at lower energies suggests stable adsorption sites where KEDG molecules are likely to bind strongly. The narrow peaks indicate that most adsorption configurations have similar energies, suggesting uniformity in binding strength at those sites.

Thus, the energy values are in agreement with standard free energy of adsorption ($\Delta G_{ads}^{o}$) obtained from the Freundlich isotherm (−30.86 kJ mol^−1^), confirming the physical adsorption mechanism accompanied by the chemical-moderated one.

The most thermodynamically stable adsorption configuration, Cu (1 1 1)/ KEDG, characterized by the lowest energy level of −227.029 kcal mol^−1^ (−54.26 kJ mol^−1^), indicates the parallel orientation with respect to the metal surface, the maximum adsorption distance being 10 Å.

**Figure 11:**
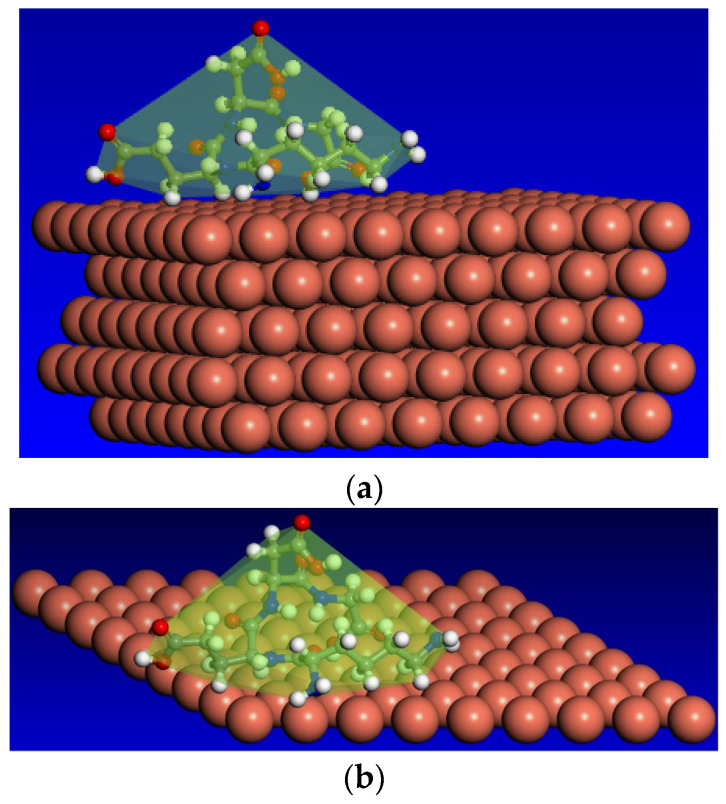
Adsorption configuration corresponding to the global minimum on the potential energy hypersurface: side view (**a**); top view (**b**).

The images obtained through an iterative procedure are presented in Figure 11.

The negative value of the adsorption energy indicates the spontaneity and exothermicity of the KEDG attachment on the copper surface, and by magnitude, it highlights the strength of the interactions between KEDG and the Cu (1 1 1) surface.

**Figure 12:**
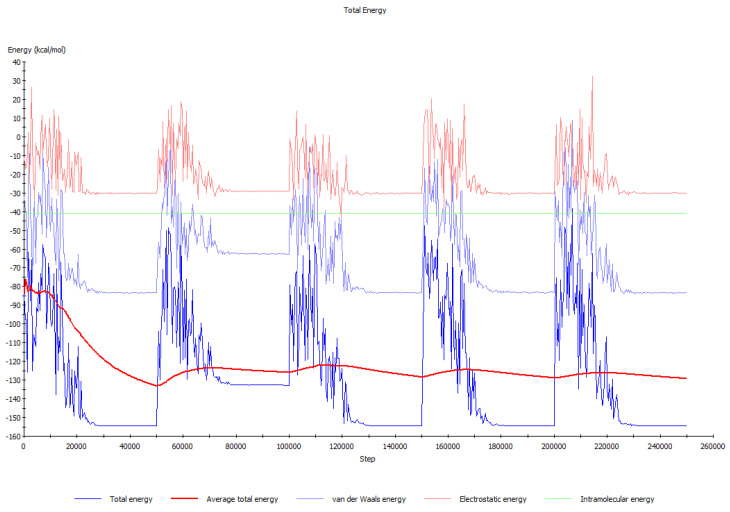
Total energy distribution profile of the Cu (1 1 1)/Lys-Glu-Asp-Gly system.

The profile of the total energy curve (Figure 12) includes the contributions of different interactions such as van der Waals forces, electrostatic interactions, and chemical bonds, as well as the adsorption distance maximum. Thus, it can be stated that the adsorption equilibrium establishment is the consequence of the simultaneous contribution of noncovalent (electrostatic and van der Waals) and covalent interactions.

**Figure 13:**
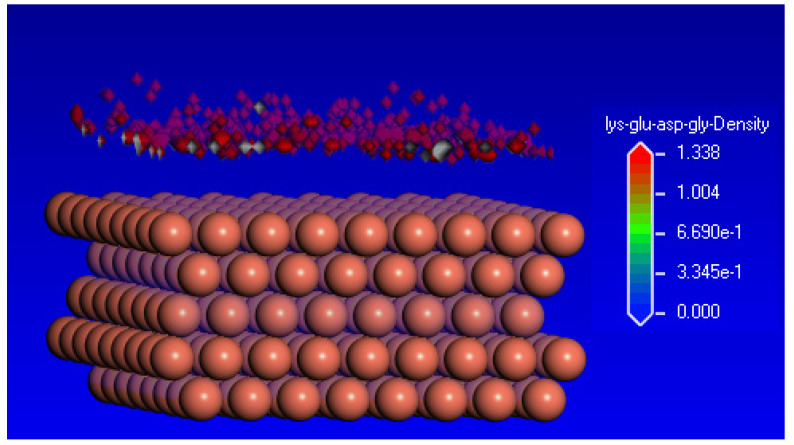
Volumetric representation of the spatial density distribution of the KEDG on the Cu(1 1 1) surface.

Monte Carlo simulation of the interaction between the KEDG tetrapeptide and the Cu(1 1 1) surface also allowed the calculation of the density field by generating a three-dimensional grid on the metal surface where the density values were determined based on the position of the adsorbate molecules during the simulation runs of the adsorption scenarios. The hypothesis of the high adsorption capacity on the Cu(1 1 1) surface of the KEDG is confirmed by the volumetric representation of the density field (Figure 13).

The high-density areas indicate strong interactions between the KEDG and specific sites on the surface, which are associated with the highly negatively charged heteroatoms in the Lys-Glu-Asp-Gly molecule, highlighted by the DFT calculation of the natural atomic charges.

Note that the KEDG computational study did not consider the effect of Cl^−^ ions from the electrolyte that can influence quantum chemical calculations and molecular dynamics of KEDG in the studied system. Thus, as a perspective, the influence of the corrosive environment composition constitutes a challenge for DFT calculations and Monte Carlo simulation.

The chloride ions can have a minimal impact on the values of the KEDG global descriptors. However, the KEDG main characteristics, such as the energy gap between the frontier molecular orbitals LUMO and HOMO, the distribution and mapping of HOMO and LUMO, the molecular electrostatic potential, the local ionization potential, the assignment of natural charges to the atoms inside the KEDG molecule and the dipole moment, remain almost unchanged. These can fall within the tolerable errors that can also occur between the studies reported by different authors. Also, the KEDG action mechanism is carried out through the similar active centers belonging to its molecule. Therefore, the same noncovalent (electrostatic, van der Waals) and covalent (chemical) interactions occur between inhibitor and copper surface, leading to KEDG adsorption.

### 2.6. KEDG Action Mechanism

According to experimental data computed from the adsorption isotherm, the Freundlich factor (n) reached a value of 2.58, indicating that KEDG adsorption is favorable, the peptide having a good affinity for the copper surface, which was also demonstrated by the theoretical calculations. The standard free energy of adsorption ($\Delta G_{ads}^{o}$) value of −30.86 kJ mol^−1^ shows that the KEDG attachment on the copper surface is spontaneous, suggesting the physical adsorption process is accompanied by a moderate chemical one.

Theoretical calculations show that KEDG can interact with the metallic surface either by electrostatic bonds or by donating electrons to the copper surface. The adsorption process is facilitated by the oxygen and nitrogen atoms belonging to the KEDG side chain as well as to the peptide groups. Also, the electronic cloud extended to certain carbon atoms can stimulate the adsorption process. On the other hand, the organic molecules inhibit the metal corrosion acting by adsorption on the oxide layer/copper surface, usually configuring certain chelates/complexes [50]. Consequently, it can be estimated that KEDG donates electrons to the copper ions from the electric double layer, thus forming KEDG–copper chelates/complexes, which can bind to the substrate through van der Waals forces.

Consequently, the KEDG physical adsorption can take place through noncovalent bonds such as the van der Waals forces and electrostatic interactions between partially charged centers of the peptide molecular chain and the charged copper surface supplemented by the chemical one occurring by covalent interactions.

Generally, the vacant *d*-orbital of transition metals allows molecule parallel chemisorption by an accentuated π-d orbital hybridization or perpendicular chemisorption occurring through σ-molecular orbitals of unsaturated heteroatoms.

In the case of copper’s completely occupied *d*-orbitals, it is possible that moderate perpendicular chemisorption of KEDG through σ-molecular orbitals can take place [50,51]. This hypothesis does not contradict the Monte Carlo simulation indicating the parallel orientation of KEDG molecules with the copper surface, the chemical interactions taking place between unsaturated oxygen atoms of the tetrapeptide molecules and copper cations from the electric double layer, forming complexes which are oriented parallel to the metallic surface, attaching to it through van der Waals forces.

The KEDG molecule regions susceptible to easily donate electrons to the copper ions from the electric double layer were also identified by the local ionization potential map simulation, as shown in Figure 8b.

## 3. Materials and Methods

### 3.1. Materials

Plates with an area of 1 cm^2^ were cut from a copper foil (thickness of 0.3 mm) with a purity > 99% purchased from a local supplier, Craiova, Romania, were subjected to corrosion in 0.9% NaCl solution in the absence and presence of a potential corrosion inhibitor. In order to delay copper corrosion in NaCl solution, a tetrapeptide based on lysine, glutamic acid, aspartic acid, and glycine (Lys-Glu-Asp-Gly), known as testagen or KEDG, was investigated.

The tetrapeptide was supplied in 20 mg vials by Pen peptides, a Romanian supplier that purchased it from DTS Pharmacy LTD, Lithuania. According to the analysis certificate, KEDG is a white powder with a purity of 98.44%. The molecular formulas of the constituent amino acids of KEDG and its optimized structure (the last being achieved within this study) are presented in Scheme 1.

Bi-distilled water and NaCl with analytical purity, obtained from Merk, were used to prepare the corrosive media, as follows: (1) 0.9% NaCl solution, hereinafter referred to NaCl blank solution (without KEDG); (2) 0.9% NaCl solution containing various KEDG concentrations: 5 mg L^−1^; 10 mg L^−1^; 15 mg L^−1^; 20 mg L^−1^.

### 3.2. Methods

#### 3.2.1. Electrochemical Measurements

The electrochemical measurements of OCP and potentiodynamic polarization were carried out on copper plates in 0.9% NaCl solution at room temperature. An electrochemical cell with three electrodes coupled to a potentiostat/galvanostat VoltaLab 40 with VoltaMaster 4 software (corrosion option) was used.

The copper plate with area of 1.0 cm^2^ constituted the working electrode, while a similar platinum one was used as the auxiliary electrode, and an Ag/AgCl electrode was connected as a reference electrode. In our early studies, a similar electrochemical system was employed to investigate the metal corrosion inhibition in different media [2,21,30]. Before the corrosion tests, the copper specimens were polished with emery paper having different sizes of abrasive particles, from grits of 120 and 220 for rough cleaning to grits of 400 and 800 for effective finishing. After mechanical treatment, the copper samples were ultrasonically cleaned, degreased with acetone, and dried in warm air.

The classical potentiometry was carried out in a potential range from −550.0 mV to 150.0 mV, with a potential scan rate of 1.0 mV s^−1^, after 4.0 min from immersing the electrodes in NaCl solution, in the absence and presence of KEDG.

In order to compute the electrochemical parameters, the potentiodynamic curves, i = f(E), were recorded and processed as semilogarithmic diagrams, in the potential range of ± 200 mV, in respect to the corrosion potential. Also, the corrosion rate (CR) was simultaneously calculated and displayed with the electrochemical parameters. To determine the corrosion rate (CR), the potentiogravimetric method was applied by VoltaMaster 4 software that allows the conversion of i_corr_ (μA cm^−2^) to CR (μm Year^−1^), using Equation (10) introduced in Section 2.1.4.

Also, the linear diagram was recorded at potentials very close to the corrosion potential for overvoltages ± 10 mV, on one side and the other of E_corr_.

The data were collected from five experiments performed for each inhibitor concentration as well as for blank NaCl solution. The sets of experimental data were analyzed in Excel by successively accessing (1) data; (2) data analysis; (2a) descriptive statistics; (2b) summary statistics. A confidence level for the mean between 95% and 97% was processed. Therefore, the standard deviation is provided and listed in Table 1 and Table 2.

#### 3.2.2. Surface Characterization

Optical microscopy and SEM/EDS analysis were used to examine the copper surface morphology before and after corrosion in NaCl blank solution and in NaCl solution containing KEDG.

##### Optical Microscopy

Optical microscopy images were acquired for the copper specimens before corrosion and after potentiometry, respectively, as follows: (a) copper surface before corrosion (Cu_standard); (b) copper surface immersed in NaCl blank solution (Cu/NaCl); (c) copper surface immersed in NaCl containing 15 mg L^−1^ KEDG (Cu/NaCl/15 mg KEDG); (d) copper surface immersed in NaCl containing 20 mg L^−1^ KEDG (Cu/NaCl/20 mg KEDG).

The metallographic microscope, EUROMEX, with Canon camera and included software, was used as was reported in our previous studies [21,29,30]. The microscope characteristics to collect the surface images were established as follows: eyepiece magnification power, 10; lens magnification power, 40; magnifying power of the microscope, 400.

##### SEM/EDS Analysis

The morphology of the samples was evidenced by scanning electron microscopy (SEM) (SU8010 from Hitachi, Tokyo, Japan) while the chemical composition was determined as a function of the carbon baseline using an energy-dispersive X-ray spectrometer (EDXS, Oxford Instruments, High Wycombe, UK) coupled with SEM.

SEM images were collected at different resolutions and magnifications before and after copper corrosion in 0.9% NaCl solution, in the absence and presence of 20 mg L^−1^ KEDG. EDS analysis was performed for the same previously mentioned samples, hereinafter called Cu_standard, Cu/NaCl, and Cu/NaCl/20 mg L^−1^ KEDG.

### 3.3. Computational Study

#### 3.3.1. Computational Details Regarding DFT Calculations

The Density Functional Theory (DFT) model, based on the Hohenberg–Kohn theorems, was used to perform the computational calculation in the aqueous phase by simulating the continuum dielectric medium using the SM8 model. The DFT calculation of the equilibrium geometry of KEDG in the singlet fundamental state (Scheme 1b) was performed using the hybrid model of exchange-correlation functional B3LYP (Becke 3–Lee-Yang-Parr).

This expression represents a mixing result of the exchange functional containing Becke’s three parameters with the Lee-Yang-Parr correlation functional.

In the atomistic simulation, the split-valence Pople basis set was used to represent the wave function, with the valence orbitals being doubled (double-zeta) to ensure anisotropy in the atomic orbitals to form the molecular orbitals. The flexibility of the basis set was achieved by supplementing it with diffuse and polarization functions, only for atoms with Z > 1; the augmentation of the basis set is highlighted by the notation 6-31 + G (d).

The geometric optimization without symmetry restriction was performed in Cartesian coordinates at the Restricted Hartree–Fock (RHF) level. The iterative energy minimization calculation of the KEDG was performed using the Pulay DIIS (Direct Inversion in the Iterative Subspace) procedure, the selected optimization algorithm being GDM (Geometric Direct Minimization). The acceleration of convergence was achieved using the Broyden–Fletcher–Goldfarb–Shanno (BFGS) approach in the iterative subspace.

The stationary point identified on the multidimensional potential energy surface was validated as a local minimum by calculating the frequencies corresponding to the vibration modes, with only numerical values belonging to the set of real numbers being obtained; all the eigenvalues of the Hessian matrix were positive, which implies, considering the physical significance of the elements of this matrix, only positive values of the force constants. The vibrational analysis calculations were performed at the same level of theory used in the geometric optimization calculation.

#### 3.3.2. Computational Details of Atomistic Monte Carlo Simulation

The molecular-level investigation of the adsorption equilibrium of the KEDG on the copper surface was performed by gas-phase Monte Carlo simulation, involving a computational scheme based on a Metropolis-type algorithm. In the simulated annealing, 5 heating cycles with 50,000 steps for each temperature were performed.

The simulation of the face-centered cubic crystal system of copper was performed by selecting the crystallographic plane positioned with the Miller indices (1 1 1), which is associated with the highest surface atomic density, high stability, and low surface energy.

The copper surface model with the crystallographic orientation (1 1 1) was built with 5 atomic layers, each of which contained 64 copper atoms. By the lateral extension of the unitary cell, the 2D periodic model was obtained, consisting of 64 unitary cells, thus avoiding artificial interactions between the KEDG molecule and its periodic images during the optimization step.

## 4. Conclusions

To study the adsorption properties of KEDG and its inhibitory effect on copper corrosion, a specific research design based on electrochemical measurements, optical microscopy, and SEM/EDS analysis associated with the computational study involving the DFT method and Monte Carlo simulation was approached.

The potentiodynamic polarization showed that KEDG behaves as a predominantly anodic corrosion inhibitor, acting by adsorption on the copper surface and reaching an inhibition efficiency of around 86%.

KEDG adsorption on the copper surface respected the Freundlich isotherm, and the standard free energy of adsorption ($\Delta G_{ads}^{o}$) reached the value of −30.86 kJ mol^−1^. This indicated an action mixed mechanism consisting of physical and chemical spontaneous adsorption.

SEM images showed a distinct morphology for the copper surfaces before and after corrosion in 0.9% NaCl solution, in the absence and presence of KEDG, as follows: (i) changes in surface morphology of the corroded sample in the KEDG absence were highlighted, a completely distorted metallic network by the saline solution corrosive attack being profiled compared with the standard sample; (ii) in the presence of KEDG, the SEM images show the occurrence of some formations with the appearance of needle-shaped blooms coherently organized on the surface.

The EDS analysis showed that (i) in the absence of KEDG, sodium and chloride ions as well as the oxygen from CuO were randomly electrodeposited on the copper surface; (ii) in the presence of KEDG, the amount of NaCl and CuO decreased due to the inhibitor insertion improving the uniformity and adherence of the surface layer, leading to the copper oxidation restriction and its corrosion retardation, respectively.

The computational study was in full agreement with the experimental data, confirming the synergy between the physical and chemical adsorption of KEDG on the copper surface, at the same time indicating the parallel orientation of the KEDG molecules with respect to the metal surface.

The reactivity global descriptors revealed a good adsorption ability of KEDG on the copper surface, participating in the electron acceptance–donation processes. The nucleophilic character of KEDG was thermodynamically favored, prevailing over the electrophilic one. Also, strong interactions between KEDG and the copper active surface sites occurred due to the highly negatively charged heteroatoms from the KEDG molecules, as evidenced by the DFT calculation of the natural atomic charges.

Monte Carlo simulation confirmed the experimental results, showing that the KEDG adsorption on the copper surface is a consequence of the simultaneous contribution of physical (electrostatic and van der Waals) and chemical interactions.

## Figures and Tables

**Table 1:** Results of OCP measurements and repassivation current density after an immersion time of the electrodes at OCP of 4 min.

C-KEDG (mg L^−1^)	OCP ± SD (mV vs. Ag/AgCl)	i_rp_ ± SD (mA cm^−2^)
0	−180.3 ± 4.3	7.81 ± 0.4
5	−169.4 ± 1.5	7.25 ± 0.4
10	−168.8 ± 1.1	6.98 ± 0.2
15	−168.1 ± 1.0	6.67 ± 0.5
20	−159.9 ± 1.0	5.81 ± 0.6

**Table 2:** Electrochemical parameters and inhibition efficiency computed from Tafel polarization and linear diagram. The potentiogravimetric results are also displayed.

C-KEDG (mg L^−1^)	E_corr_ (mV vs. Ag/AgCl) ± SD *	i_corr_ (μA cm^−2^) ± SD	b_a_ (mV dec^−1^)	|b_c_| (mV dec^−1^)	R_p_ (kΩ cm^2^) ± SD	IE (%)	
						from Equation (8) ± SD	from Equation (9) ± SD
0	−198.5 ± 2.5	5.96 ± 1.14	57.4	96.8	2.15 ± 0.15	-	-
5	−194.5 ± 2.8	2.99 ± 1.07	56.1	95.4	4.23 ± 0.35	49.8 ± 4.2	49.2 ± 4.6
10	−185.4 ± 2.0	2.46 ± 0.36	52.3	86.8	5.2 ± 0.6	58.7 ± 1.8	58.6 ± 1.4
15	−174.5 ± 2.0	1.86 ± 0.32	49.2	80.1	7.03 ± 1	69.1 ± 3.9	69.4 ± 2.4
20	−176.2 ± 2.0	0.85 ± 0.21	48.8	79.8	15.1 ± 0.4	85.7 ± 0.9	85.8 ± 0.6
Potentiogravimetric results							
C-KEDG (mg L^−1^)		CR ± SD (μm Year^−1^)		IE (%) ± SD		θ ± SD	
0		69.57 ± 10.33		-		-	
5		35.08 ± 8.27		49.6 ± 4.5		0.496 ± 0.045	
10		28.56 ± 5.83		58.9 ± 2.2		0.589 ± 0.022	
15		19.61 ± 5.13		71.8 ± 3.7		0.718 ± 0.037	
20		9.97 ± 2.13		85.7 ± 1.0		0.857 ± 0.01	

* SD—standard deviation.

**Table 3:** The results obtained by applying some isotherms for KEDG adsorption on copper surfaces in NaCl solution.

Adsorption Isotherm	Linearized Form	Linearization Functions	Slope	Intercept	R^2^
Langmuir [37]	$\frac{C}{\theta }=C+\frac{1}{K}$	C/θ = f (C)	1	1/K	
			0.8738	6.9	0.9496
	$\frac{\theta }{1-\theta }=KC$	[θ/(1 − θ)] = f(C)	K	0	
			0.3228	−1.2955	0.8452
Freundlich [38]	$ln\theta =\frac{1}{n}lnC+lnK$	lnθ = f(lnC)	1/n	lnK	R^2^
			0.3863	−1.3537	0.9628
Flory–Huggins [39]	$ln\frac{\theta }{C}=bln\left(1-\theta \right)+lnK$	ln(θ/C) = f[ln(1 − θ)]	b	lnK	R^2^
			0.5645	−2.1572	0.7043
Temkin [40]	$\theta =\frac{1}{f}lnC+\frac{1}{f}lnK$	θ = f(lnC)	1/f	$\frac{1}{f}lnK$	R^2^
			0.2505	0.0628	0.9199
			0.5645	−2.1572	0.7043
El-Awady’s model [41]	$ln\frac{\theta }{1-\theta }=ylnC+lnK$	$ln\frac{\theta }{1-\theta }$ = f(lnC)	y	lnK	R^2^
			1.2164	−2.1573	0.8665
Frumkin [39]	$ln[C^{-1}\left(\frac{\theta }{1-\theta }\right)]=2\alpha \theta +lnK$	$ln{[C}^{-1}(\frac{\theta }{1-\theta })]=f(\theta )$	2α	lnK	R^2^
			NA		

**Table 4:** $\Delta G_{ads}^{o}$ and adsorption parameters of KEDG on the copper surface computed from the Freundlich equation.

Freundlich Adsorption Parameters	1/n	n	lnK	K (L mg^−1^)	${\Delta G}_{ads}^{o}$ (kJ mol^−1^)
	0.3863	2.58	−1.3537	0.2583	−30.86

**Table 5:** Reactivity global descriptors of KEDG compared with those of glutathione, glutamic acid, and glycine.

Amino Acid/ Peptide	E_HOMO_ (eV)	E_LUMO_ (eV)	ΔE (eV)	I (eV)	A (eV)	χ (eV)	ε (eV)	η (eV)	σ (eV)^−1^	ΔN	ω (eV)	Ref.
KEDG/Cu(1 1 1)	6.61	−1.14	5.47	6.61	1.14	4.025	−4.025	2.885	0.347	0.159	2.808	This study
Glutathione/Cu(1 1 1)	6.84	−1.24	5.61	6.84	1.24	4.04	-	2.80	0.36	0.14	2.91	[3]
Glutamic acid/Cu(1 1 1)	7.32	−1.05	6.27	7.32	1.05	4.18	-	3.13	0.32	0.1	2.79	[3]
Glycine/Cu(1 1 1)	7.39	−0.92	6.47	7.39	0.92	4.15	-	3.24	0.31	0.1	2.66	[3]

## Data Availability

All of the data are available in the main text of the article.

## Abbreviations

The following abbreviations are used in this manuscript:

KEDG	testagen tetrapeptide
OCP	open circuit potential
i_corr_	corrosion current density
E_corr_	corrosion potential
R_p_	polarization resistance
η	overvoltage
CR	corrosion rate
b_a_ and b_c_	anodic and cathodic Tafel slopes
IE%	inhibition efficiency
K	adsorption–desorption constant
$\Delta G_{\mathrm{ads}}^{o}$	standard free adsorption energy
θ	surface coverage degree
DFT	Density Functional Theory
χ	electronegativity
ε	chemical potential
η	hardness
σ	softness
ω	electrophilicity index
ω	electrodonating power
ω+	electroaccepting power
ΔN	fraction of the transferred electron
